# Towards a data-driven characterization of behavioral changes induced by the seasonal flu

**DOI:** 10.1371/journal.pcbi.1007879

**Published:** 2020-05-13

**Authors:** Nicolò Gozzi, Daniela Perrotta, Daniela Paolotti, Nicola Perra

**Affiliations:** 1 Networks and Urban Systems Centre, University of Greenwich, London, United Kingdom; 2 Max Planck Institute for Demographic Research, Rostock, Germany; 3 ISI Foundation, Turin, Italy; Institute for Disease Modeling, UNITED STATES

## Abstract

In this work, we aim to determine the main factors driving self-initiated behavioral changes during the seasonal flu. To this end, we designed and deployed a questionnaire via Influweb, a Web platform for participatory surveillance in Italy, during the 2017 − 18 and 2018 − 19 seasons. We collected 599 surveys completed by 434 users. The data provide socio-demographic information, level of concerns about the flu, past experience with illnesses, and the type of behavioral changes voluntarily implemented by each participant. We describe each response with a set of features and divide them in three target categories. These describe those that report i) no (26%), ii) only moderately (36%), iii) significant (38%) changes in behaviors. In these settings, we adopt machine learning algorithms to investigate the extent to which target variables can be predicted by looking only at the set of features. Notably, 66% of the samples in the category describing more significant changes in behaviors are correctly classified through Gradient Boosted Trees. Furthermore, we investigate the importance of each feature in the classification task and uncover complex relationships between individuals’ characteristics and their attitude towards behavioral change. We find that intensity, recency of past illnesses, perceived susceptibility to and perceived severity of an infection are the most significant features in the classification task and are associated to significant changes in behaviors. Overall, the research contributes to the small set of empirical studies devoted to the data-driven characterization of behavioral changes induced by infectious diseases.

## Author summary

Human behavior and infectious diseases are linked by a feedback loop. While individuals might change their behavior as a response to an epidemic, such changes might influence the spreading itself. So far, our understanding and characterization of behavioral changes induced by diseases has been strongly limited by the lack of empirical data. As result, the vast majority of research has been focused on theoretical, what if, scenarios. In this work, we collected a unique dataset comprised of 599 surveys submitted by 434 users to the participatory surveillance platform Influweb over the 2017 − 18 and 2018 − 19 flu seasons. The data provide socio-demographic information, level of concerns about the flu, past experience with illnesses, and the type of self-initiated behavioral changes implemented by each participant. Our analysis, conducted adopting machine learning algorithms, show that both past experience of illness and personal beliefs about the disease are fundamental drivers of behavioral change. These findings are in good agreement with the constructs of the Health Belief Model and provide, to the best of our knowledge, the first data driven characterization of behavioral changes during the seasonal flu.

## Introduction

Understanding and influencing behavioral changes are key challenges for a range of disciplines such as Medicine, Psychology, Epidemiology, Social Policy and Computational Social Science. Most of the relevant literature focuses on *complex interventions* designed to nudge populations to adopt healthier and safer habits. Recommendations about the increase of physical activity [1], quit smoking [2], change of behaviors in workplaces [3], or promoting safe sexual behaviors [4] are classic examples. Yet, even in the absence of top-down (complex) interventions, external incentives, or penalties, people may spontaneously modify their behaviors in response to different types of events. Infectious diseases are a notable example of both [5–10]. Indeed, they may induce a range of governmental (i.e. top-down) and/or self-initiated (i.e. bottom-up) (re)actions such as social distancing (e.g., reduction of contacts or mobility, self-isolation, quarantine, closure of public places, bans of gatherings), use of antivirals, change of diets and of personal hygiene practices [6].

As we write, COVID-19 is sweeping the world with unprecedented socio-economical costs. The virus has tragically exposed our unpreparedness to deal with emerging diseases. Furthermore, it has highlighted how developing an understanding of behavioral changes is crucial to i) increase the predictive power and the realism of epidemic models, ii) improve communication campaigns from a public health perspective, iii) improve our understanding of human dynamics under stress. In fact, human dynamics and human transmissible diseases are intertwined: an outbreak can induce behavioral responses which in turn can affect the course of the epidemic as a whole [11–22]. While this observation is rather obvious, our understanding of behavioral changes is extremely limited and largely anecdotal [6, 11, 12]. Arguably, the key issue is the lack of ground truth data. According to a recent review, only 15% of the articles on the subject considered empirical data, most models being “purely theoretical and lack(ing) representative data and a validation process” [6]. The mobilisation of several communities to develop tools aimed at understanding and modeling changes in behaviors induced by the COVID-19 pandemic provides further evidence of such limitations [23–28]. Surveys represent the most common data source for these research efforts [25–27, 29–34]. Indeed, they allow to gather ground truth data, querying participants with specific questions about changes in behaviors. However, they are limited by small sample sizes, subjectivity of participants in interpreting questions, recall their actions and thought processes in the past. An increasing number of works exploit social media data and other digital sources of information to characterize the behavioral response of humans to the spreading of infectious diseases [10, 30, 35–39]. While this data allows to drastically increase the sample and to collect information in near real time (which is particularly important during the unfolding of an outbreak), the ground truth is typically missing. Thus, a set of assumptions are needed to connect the online (i.e. people’s posts) and offline worlds (i.e. behavioral changes).

In this context, we aim at advancing our comprehension of self-initiated, bottom-up, behavioral changes induced by infectious diseases with an approach that puts ground truth data about individuals’ behaviors at its core. As an example of recurring and widely spread human transmittable disease, we consider seasonal influenza. The reasons behind this choice are four. First, the World Health Organization, estimates the seasonal flu to result in about 3 to 5 million cases of severe illness, and about 290, 000 to 650, 000 respiratory deaths worldwide [40]. Besides the cost in terms of human lives, the seasonal flu represents also one of the main economic costs for public health systems [41]. Second, the seasonal flu is transmitted via droplets, droplets nuclei (i.e. aerosols) and contacts [42, 43]. Thus, the routes of transmissions are intimately linked and affected by our behaviors; from social interactions to hygiene standards [44, 45]. Third, we can leverage existing participatory Web platforms for digital surveillance of the seasonal flu to reach and query large numbers of users with explicit questions about behavioral changes. In fact, the yearly cadence of the seasonal flu allows the planning of regular data collection campaigns that can go beyond gathering information about disease’s prevalence. An important behavioral aspect that participatory surveillance already tackles is the healthcare seeking one. During seasonal influenza periods, the majority of individuals with symptoms don’t go visit a doctor. Having a way to ask people about their symptoms that are not detected by traditional surveillance methods is important to shed a light on this individual behavior that affects the accuracy of the epidemiological signal. But going beyond symptoms, as we do here, can provide unprecedented insights on spontaneous changes in social norms that can have an impact on how diseases spread. This is becoming crucial especially now, with the CODIV-19 pandemic, when even self-implemented changes can dramatically slow down the diffusion of the disease. Fourth, the few empirical studies on behavioral changes induced by infectious diseases are mostly focused on pandemics such as the 1918 Spanish flu [46–48], the 2009 swine flu [8–10, 29, 33, 34], and as we write, COVID-19 [23–28]. However, these events are rare; their timing, intensity, patterns, media coverage, and governmental interventions are often out of the ordinary. The COVID-19 pandemic unfortunately offers a vivid example. At the moment of writing, as a way to slow-down the spreading, many countries have implemented measures that range from closing school, bars, restaurants and public gatherings to more strict nation-wide lock-downs. In absence of pharmacological solutions social distancing is the only option. These measures are unprecedented and the resulting drastic changes in behaviors are, to different extents across countries, not self-initiated but mandated top-down interventions. Thus, the relevance of existing and future literature focused on pandemics for other outbreaks and diseases is unclear.

In this work, we combine health and behavioral data, collected from Web users, with a machine learning pipeline to characterize self-initiated behavioral changes during the seasonal flu. In particular, we developed and deployed a questionnaire via Influweb [49, 50], a digital surveillance platform that since 2008 collects data about the progression of the seasonal flu in Italy, to collect socio-demographic indicators, medical history of individuals, information regarding feelings, concerns towards the flu and to query users about changes in their behaviors induced by the disease. By studying the responses, we identify three classes of behavioral changes describing those that report i) no (26%), ii) only moderately (36%), iii) significant (38%) changes in behaviors. From this standpoint, we adopt a range of machine learning algorithms such as Gradient Boosted Trees (GBT) [51], Support Vector Machine (SVM) [52], Logistic Regression (LG) [53] and Random Forest (RF) [54] to solve a classification task in which a set of 23 features (obtained from the responses and the characteristics of the epidemic) are used to predict the class of behavioral change of each user. In order to interpret the outcomes of the classifiers, we use SHapley Additive exPlanations (SHAP) values [55–57]. These allow to measure the importance of each feature in the classification task. We find that GBT is able to correctly classify 66% of the samples describing significant changes. Interestingly, our result indicate that the severity and recency of past illnesses, the perceived susceptibility to the disease, the perceived severity of infection events are the key factors driving behavioral changes. The last two drivers are in line with the constructs of the Health Belief Model (HBM) [58–60], which is by far the most commonly used psychological theory to explain and predict health-related behaviors. Furthermore, we find that the progression of the disease, the information collected about it, the risks to affect vulnerable others are also relevant factors influencing behavioral changes.

Overall, these results quantify the extent to which individuals voluntarily change behaviors in response to the seasonal flu, uncover the key factors influencing such changes, and quantify the limits of predictability of behavioral classes in our sample. The research presented here contributes to the unfortunately still small set of empirical data-driven studies on disease outbreaks and behavioral changes. To the best of our knowledge, this is the first data-driven study focused on the seasonal flu and the first to use data on behavioral changes, induced by diseases, collected from a digital surveillance platform. The methodology and findings presented here pave the way to future extensions and generalizations able to capture multiple diseases, larger sample sizes as well as different countries. This methodology potentially represents a public health monitoring tool. In fact, the routine surveillance from Influweb are already communicated to the Italian National Institute of Public Health every week during the influenza season since 2015. Additional quantitative assessment about the change in behaviors induced by the flu among the general population could represent a valuable insight for policy makers when communicating recommended behaviors to avoid contagion.

Finally, as of April 2020, the COVID-19 pandemic is already set to be a defining moment of the decade, if not more. It is hard to immagine what the long lasting impact of the crisis on our socio-economic fabric will be. Arguably however, the public has been sensibilized, as never before, to the importance of simple hygiene measures, such as washing hands, to reduce the chances of transmission and of social distancing as key weapon against transmittable diseases. It is natural to wonder how these unprecedented events will affect our behaviors during future outbreaks such as the next flu seasons. Answers to this question might be found by comparing our findings (pre-pandemic) with similar future studies (post-pandemic). Thus, beside the specific contributions highlighted above, our paper has the potential to serve as time capsule, informing later efforts aimed at quantify the effects of COVID-19 on our (re)actions to coming outbreaks.

## Materials and methods

### Influweb dataset

Influweb is a scientific project aimed at monitoring the activity of Influenza-like Illness in Italy with the aid of volunteers via the internet [49, 50]. It has been operational since 2008 and it is part of the InfluenzaNet network, active in many other European and non-European countries [61, 62], such as The Netherlands, Belgium, Portugal, United Kingdom, Sweden, France, Spain, Denmark and Ireland. Throughout the years InfluenzaNet platforms have been shown to be reliable sources of high-resolution and high-quality public health information [61, 63–65]. In this work, we focus on the data collected through the Italian node of the platform, Influweb by means of three surveys:

- *Intake questionnaire*: is submitted when the user completes the registration and can be updated at the beginning of a new season; it covers demographic, geographic, socioeconomic (household size and composition, occupation, education, and transportation), and health (vaccination, diet, pregnancy, smoking, and underlying medical conditions) indicators.
- *Symptoms questionnaire*: is submitted weekly during the flu season. Participants are asked whether they experienced fever, respiratory or gastrointestinal symptoms (or “no symptoms”) since their last survey. If symptoms are reported, further questions are asked to assess the syndrome (e.g, sudden onset of symptoms and body temperature).
- *Behavioral questionnaire*: is submitted during the flu season and contains questions related to perceptions towards the flu and behavioral attitude of participants.

The behavioral questionnaire has been designed and deployed in Influweb, as part of this study, during the 2017 − 18 and 2018 − 19 seasons, with the aim of shedding a light on behavioral aspects beyond the mere epidemiological data collection conducted via the two other questionnaires since 2008. A full account of the behavioral change questionnaire can be found on the Influweb Web page [66]. In order to reduce the burden for the users, the behavioral questionnaires have been administered only during few weeks in each season, i.e. right before the peak and a couple of weeks after the peak. We refer the reader to the Supporting Information (SI) for more details. In total 599 behavioral surveys were submitted by *N* = 434 unique users: 73% responded only once, 27% instead more than once (in the same and/or across seasons). Consequently, while the large part of surveys are uniquely linked to a specific user, some are not. It is important to notice how sentiments, perceptions, and behaviors might vary during the flu season. Thus, we consider the 599 surveys, rather than the users, the unit of analysis. As shown in the SI, this choice does not affect the overall results.

### Ethics statement

Informed consent was obtained online from all participants enabling the collection, storage, and treatment of data, and their publication in anonymized, processed, and aggregated forms for scientific purposes. The Influweb website [50] has a “Privacy Policy” section in which the users who decide to enroll in the study can find all the information on who is responsible for the data acquisition and processing.

### Behavioral change classes

**Fig 1:**
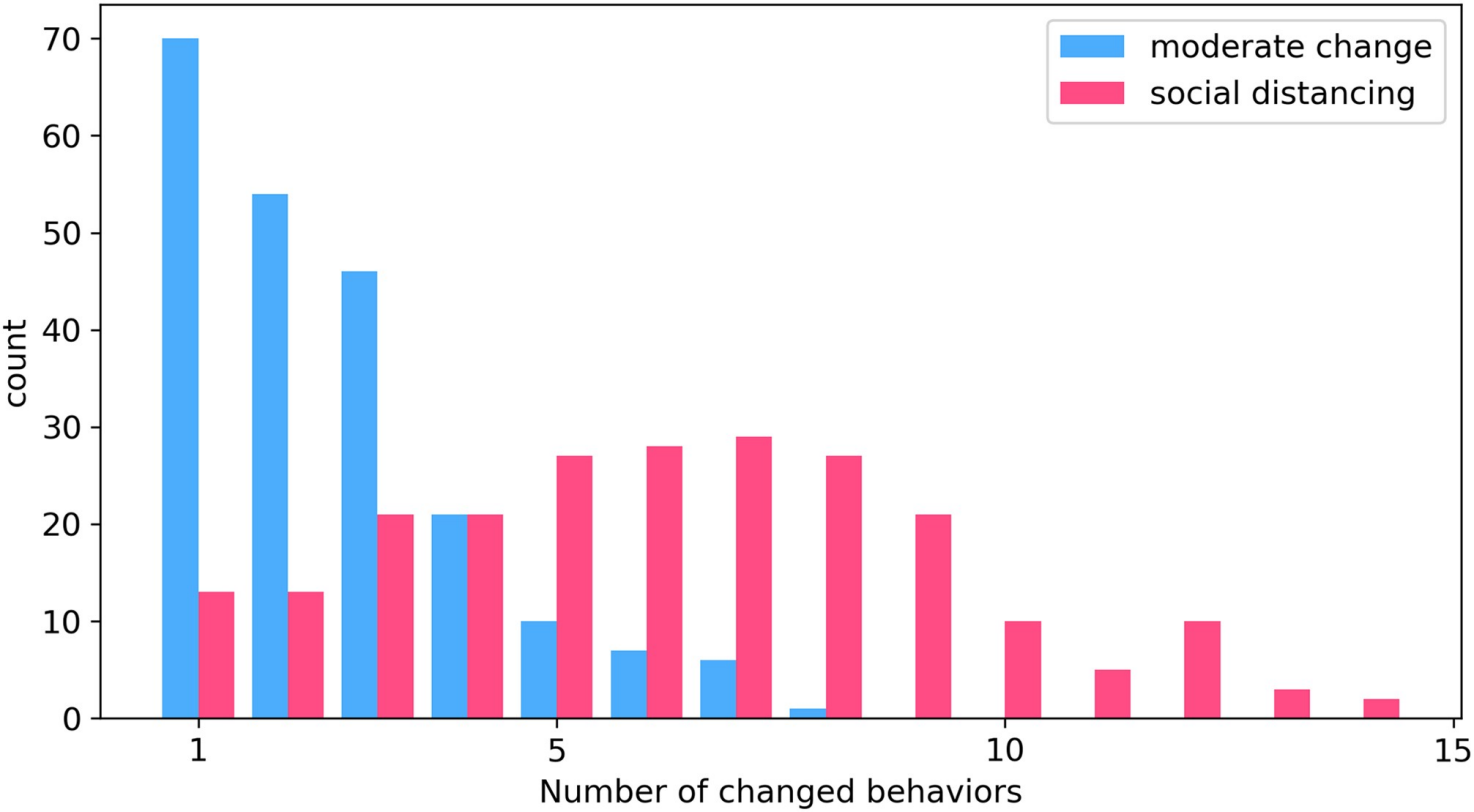
Histograms of number of behaviors changed for the class of *social distancing* and for class of *moderate change*. The two histograms look significantly different, with that of social distancing much more skewed towards higher values.

In the behavioral survey, participants were asked, among other things, whether they have changed or not a number of behaviors in response to the flu. A natural categorization of the participants’ responses would be to consider on one side individuals who do not change any of the possible behaviors, and on the other individuals who change at least one. However, our data suggest that this approach can be too restrictive. In fact, individuals who report to engage in behavioral change seem to form two different groups: 1) individuals who take only moderate preventive measures, such as more frequent hygiene measures, a healthier diet or the use of tissues when sneezing or coughing more often than usual, 2) individuals who, besides the previous precautions, take also social distancing measures as a response to the epidemic. For example, they take time off work, cancel or postpone social events, or use less public transportation. In the SI we report a list of all possible behaviors changed by individuals in these two groups. Indeed, the average number of behaviors changed by individuals who report at least one social distancing measure is much higher than the average of those who take only moderate preventive measures (respectively 6.26 and 2.52). This can be observed also in Fig 1, where we show the histograms of the number of behaviors changed in the two classes. Furthermore, individuals who report at least one social distancing measure do also report at least one moderate preventive measure. These observations support the idea that in our dataset are present at least two main forms of behavioral change: moderate preventive measure and social distancing. The latter can be regarded as a reinforcement of the former. Furthermore, the approach of dividing individuals into three classes aims at providing a more composite representation of behaviors. In ref. [67] is underlined that most of current models do not allow for heterogeneous behavioral responses to an epidemic. However, this homogeneous assumption is broadly inconsistent with what we know about human behavior [68, 69].

In summary, we divide individuals according to their responses into three classes:

- individuals who do not change their behavior (defined as *no change* in the following). This class corresponds to 26% of responses;
- individuals who take only moderate preventive measures (defined as *moderate change* in the following). This class corresponds to 36% of responses;
- individuals who take also social distancing measures (defined as *social distancing* in the following). This class corresponds to 38% of responses.

Nevertheless, in the SI we report the results of the analyses done considering only two categories: 1) change 2) no-change. The results show a classification performance significantly higher than the random benchmark, although the relative difference between the two is lower than with three categories. Most importantly, the key features for classification in the two classes are consistent with those that emerge considering a tripartite division.

### Features engineering

As mentioned above, our dataset comprises intake, symptoms, and behavioral questionnaires collected from Influweb during the 2017 − 18 and 2018 − 19 flu seasons in Italy. We combine all these data obtaining 23 features for each of the 599 responses. In particular, the features have been created based on the following information: 1) socio-demographic indicators (age, gender, etc.), 2) health indicators (allergies, chronic diseases, frequency of flu episodes, etc.), 3) information indicators (whether the user actively sought information about the flu, self-assessment of the level of information about the disease), 4) feelings and beliefs towards the flu (concerns, impact on personal life of a possible contagion, etc.), 5) epidemic indicators (incidence of flu epidemics at the moment of response and timing of the peak respect to the moment of response).

In Table 1 we provide a complete list of features with related meanings. Before moving forward, it is important to describe in some more details the construction of three key features. In particular, we define a *disease score* that aims to provide a measure of the severity of the illnesses experienced in the past by participants. We define it as:

$$\begin{array}{c} \text{disease}\;\text{score}=\sum_{i}\frac{M_{i}n_{i}e^{\gamma \Delta_{i}}}{n_{i}e^{\gamma \Delta_{i}}} \end{array}$$ (1)

Where *i* runs over all the weekly (symptoms) questionnaires submitted by the individual; *M*_*i*_ is the number of symptoms reported in the *i* − *th* weekly questionnaire; $e^{\gamma \Delta_{i}}$ weighs the duration of illness giving more importance to the most recent ones. In particular, Δ_*i*_ is the difference—in years—between the submission date of the *i* − *th* weekly and the behavioral survey, and γ is a parameter that expresses how fast people forget past experiences (here set to 1 year); *n*_*i*_ is the number of individuals present in our dataset who reported their symptoms during the same week of the *i* − *th* weekly questionnaire. Weighing observations with this term gives less importance to periods with just a few active participants and makes the *disease score* of different individuals comparable. The *disease score* can then be interpreted as follows: the higher, the more recent and severe the episode experienced by the individual. In the SI we test a much simpler definition of *disease score* where we disregard the exponential temporal weights $e^{\gamma \Delta_{i}}$. Adopting a simpler definition slightly reduces the precision, but does not change the overall results.

**Table 1:** List of features.

Type	Feature	Meaning
**socio-demographic**	*gender*	gender
	*age*	age class (15, 15-30, 30-50, 50-65, 65+ years)
	*contacts*	true if the individual has daily contacts with large groups, patients, children
	*smoke*	true if the individual smokes regularly
	*diet*	true if the individual follows a special diet
	*children*	true if the individual has children in school age
	*public transport*	true if the individual takes regularly public transportation
	*elderly*	true if the individual has old people (65+) in her household
**health-related**	*flu frequency*	frequency of flu-like illness
	*flu*	true if the individual had flu in the current season
	*disease score*	measure of severity of diseases experienced in the past
	*vaccination*	true if the individual has received a vaccine in the current season
	*vaccination last year*	true if the individual has received a vaccine in the previous season
	*allergy*	true if the individual has allergies that can cause respiratory problems
	*disease*	true if the individual receives regularly medication for chronic diseases
**information-related**	*info seeking*	true if the individual seeks regularly information regarding the flu
	*information*	self-evaluation of the level of information regarding the flu
**beliefs**	*preventive*	true if the individual thinks that proactive measures can prevent the contagion
	*perceived susceptibility*	measure of anxiety deriving from a possible contagion
	*efficacy*	measure of awareness of efficacy of behavioral measures. It can assume integer values in between 0 and +8, the higher the more the individual believes that behavioral change can lower the risk of an infection
	*perceived severity*	measure of concerns related to the possibility of contagion
**epidemic indicators**	*peak*	days between the ILI peak and the date of compilation of the behavioral survey
	*prevalence*	flu prevalence in the Italian region where the participant reside in

We then define a *perceived susceptibility* measure. Participants are asked some questions regarding their feelings and perceptions towards the flu. The reduced STAI (State-Trait Anxiety Inventory) test is used as guideline for this questionnaire [34, 70]. The goal is to assess their level of anxiety towards a possible contagion. To each of the questions they can answer either yes, no, do not know. We turn possible answers in numeric values, respectively +1, −1, 0. Then, we sum all the answers. The resulting variable can assume values between −4 (minimum level of anxiety) and +4 (maximum level of anxiety). This to quantify the fact that individuals who are more anxious at the idea of becoming infected, also perceive themselves as more susceptible to the disease.

Finally, we define a *perceived severity* measure. Participants are asked to evaluate some statements regarding the consequences of a possible contagion (for instance “Flu would be a serious illness for me” or “A contagion would have serious financial consequences for me”). This is done to asses the perceived impact that a possible contagion would have on individuals’ life in general. Participants can express their level of agreement with statements through the possible answers probably true, probably false, do not know. We turn possible answers in numeric values, respectively +1, −1, and 0 and we sum all answers to obtain the feature *perceived severity*. It can assume values between −4 (minimum perceived severity) and +4 (maximum perceived severity). To the best of our knowledge, there is not a standard psychological test to asses perceived severity (such as, for example, the STAI test). Nonetheless, we designed questions to capture the nuanced nature of this feature. Indeed, one can perceive a possible contagion as particularly severe for a variety of reasons. Similar approaches have been used in recent surveys aimed to assess the psychological response of individuals to the COVID-19 pandemic [26, 27].

It is important to notice how several features have been designed to match the constructs of the Health Belief Model (HBM) [58–60], which is by far the most commonly used psychological theory to explain and predict health-related behaviors. The underlying concept of HBM is that health behaviors are determined by personal beliefs and perceptions about the disease: the more an individual feels *threatened* by the possibility of infection, the more she will be inclined to embrace protective behaviors. More in detail, according to the HBM the *perceived threat* of an individual is determined by two main constructs: 1) *Perceived severity* refers to the individual’s belief about the severity of the disease. The HBM proposes that individuals who perceive the disease as more severe are also more keen on protecting themselves through proactive behavioral measures. Even if *perceived severity* is often based on medical information, it can also be a consequence of the beliefs a person has about the difficulties a disease would create on her life in general; 2) *Perceived susceptibility*, instead, refers to the personal evaluation of the risk of contracting the disease. According to the HBM, individuals who consider themselves more vulnerable to the disease, are also much more likely to engage health-promoting behaviors. Of course, many other factors can influence individuals’ decision-making process. In particular, the HBM suggests the existence of *modifying variables* (such as socio-demographic indicators) to explain interpersonal variability, and of endogenous events—called *cues to action*—that prompt individuals towards the acceptance of healthier behaviors. The individual should also consider that behavioral change is actually decisive to decrease risk of contagion, and that the benefits associated with change are higher than the costs.

### Classification algorithms

The data analyses are conducted with a range of machine learning algorithms. Decision tree ensembles are a powerful tool for classification tasks [71]. They consist in a set of classification trees. In fact, the underlying idea is that summing together the predictions of multiple “weak” learners, one can achieve more robust predictions than with a single “strong” learner. This general model is implemented by a great variety of algorithms, such as Gradient Boosted Trees (GBT) [51]. GBT exploits a specific training strategy called *additive training*, in which at each training step is added to the ensemble the tree that optimizes the objective function. In this work, we use *XGBoost*, an open-source software library [72, 73]. Recently, it has gained a great popularity for its speed and performance, and has become the algorithm of choice for many machine learning applications. In practice, we use this algorithm to classify individuals according to their features in the three behavioral change classes. To quantify the quality of the predictions of the GBT model we compare it to: i) a dummy classifier that generates random predictions by respecting the training set’s class distribution. This is done to assess if and to which extent the GBT model performs better than a null benchmark, ii) other standard machine learning models such as Support Vector Machine (SVM) [52], Logistic Regression (LG) [53] and Random Forest (RF) [54]. We use the *scikit-learn* [74] implementation of these algorithms and we train them fine-tuning standard parameters. We refer the interested reader to the SI for more details and the code.

### Explainability

The ultimate goal of our analysis is to determine which are the main drivers of self-initiated behavioral changes in response to epidemics and to which extent they influence people’s behavior. To achieve this, understanding the hidden patterns spotted by the machine learning classifier is essential. To interpret model’s decisions we exploit SHAP (SHapley Additive exPlanations), a unified approach that connects cooperative game theory with local explanations to explain the output of any machine learning model [55–57]. It aims at understanding the role and the significance of each feature in the model’s decisions using Shapley values. The Shapley value is a solution concept in cooperative game theory that addresses the following issue: how important is each player to the overall cooperation, and what payoff can she reasonably expect? This is very similar to the problem we are considering. In our framework, the overall cooperation is the classification task, players are the features, and the payoff of players is the importance of features for the classification performance. We refer the readers to the SI for more details.

## Results and discussion

### Classification task

After having pre-processed data and built the features, the next step of the analysis consists in training the models to classify individuals in the three classes of behavioral change. Following a common approach, we divide our dataset in training set (70%) and test set (30%). Only the training set is used to find the optimal parameters for the models, while the test set is retained to evaluate performance. We search for optimal parameters using *10-fold cross validation* over an extensive grid of candidate values. We use four different metrics (*precision*, *balanced accuracy*, *recall* and *f1 score*) to obtain a complete overview of the performance of the classification algorithm. From results in Table 2 we observe that GBT outperforms (across the four metrics) i) the trivial prediction strategy (RND), ii) other standard machine learning algorithms (SVM, RF, LG). It is important to notice how the highest precision obtained is far from 1. However, to the best of our knowledge, this is the first paper taking such approach, thus we have no previous results (i.e. benchmarks) to compare and contrast with.

**Table 2:** Classification performance.

model	precision	bal. accuracy	recall	f1 score
RND	0.343	0.335	0.334	0.335
SVM	0.519	0.503	0.500	0.504
LG	0.479	0.492	0.478	0.472
RF	0.506	0.498	0.506	0.505
GBT	**0.546**	**0.549**	**0.550**	**0.546**

**Fig 2:**
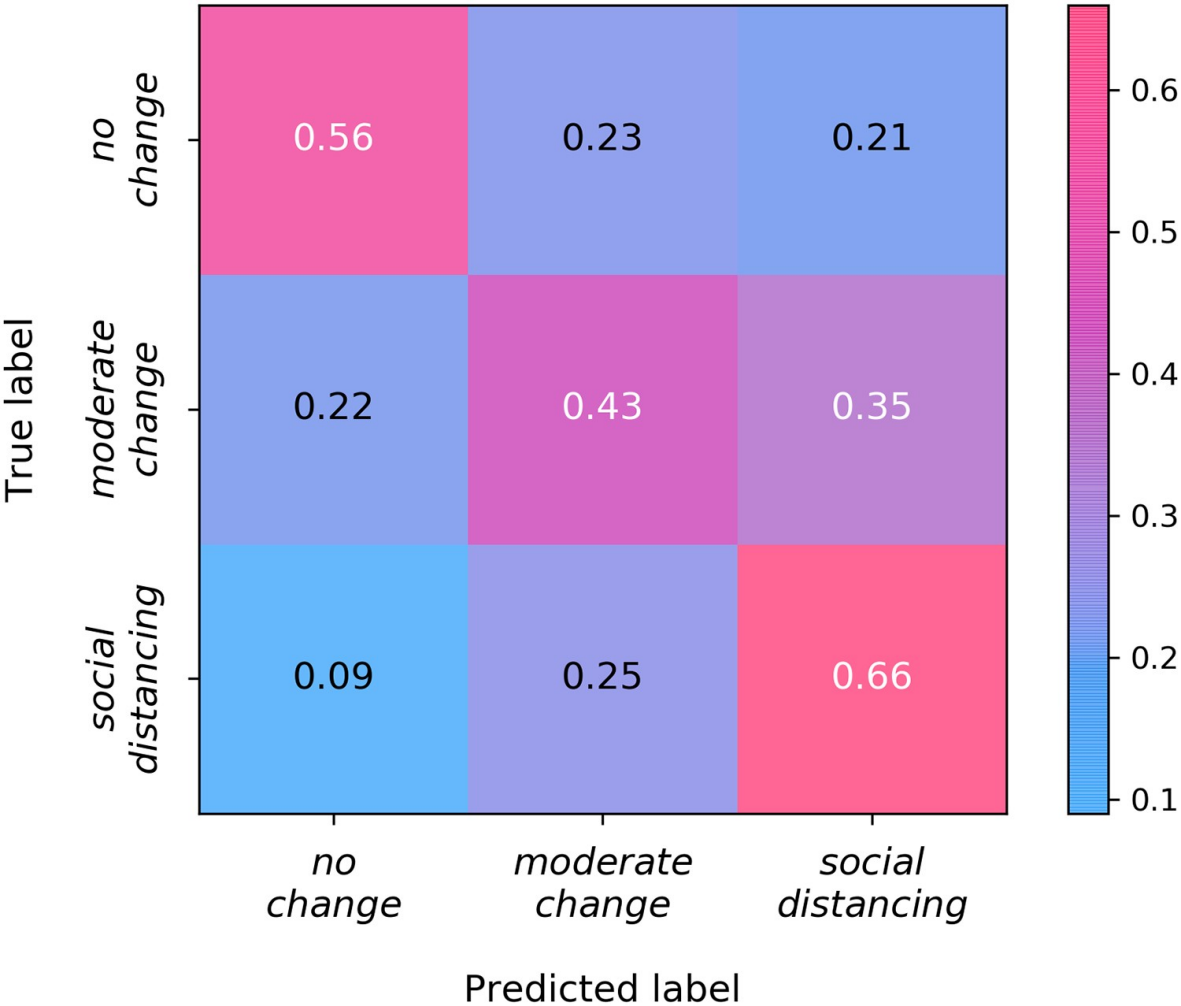
Confusion matrix of GBT. Each row and column represents a particular class: on the vertical axis are represented the true labels, while on the horizontal axis are represented the predicted labels. Hence, in the main diagonal boxes, we can observe the percentage of samples correctly labeled for each class, and in non-diagonal boxes, we can observe the percentage of misclassifications among all possible pairs of classes.

Next, we analyze in depth the performance of GBT. Fig 2 represents the confusion matrix of model’s predictions. In statistical classification problems, the confusion matrix is a specific table layout that allows visualization of the performance of an algorithm. Interestingly, the best-predicted class is social distancing. In fact, 66% of its samples are classified correctly. This result suggests that individuals changing their behaviors significantly stands out more in the feature space. Furthermore, most of the classification errors are between the two classes of behavioral change, while there are fewer errors between the two classes linked to changes in behaviors and the no change class. Thus, as one would expect, there is more similarity between responses in the two behavioral change classes than between responses of a behavioral change class and those of no change class.

### Understanding model’s decisions

In this section we want to make a step further and inspect what GBT learns from data using SHAP, the tool of explainable machine learning that we have previously introduced.

**Fig 3:**
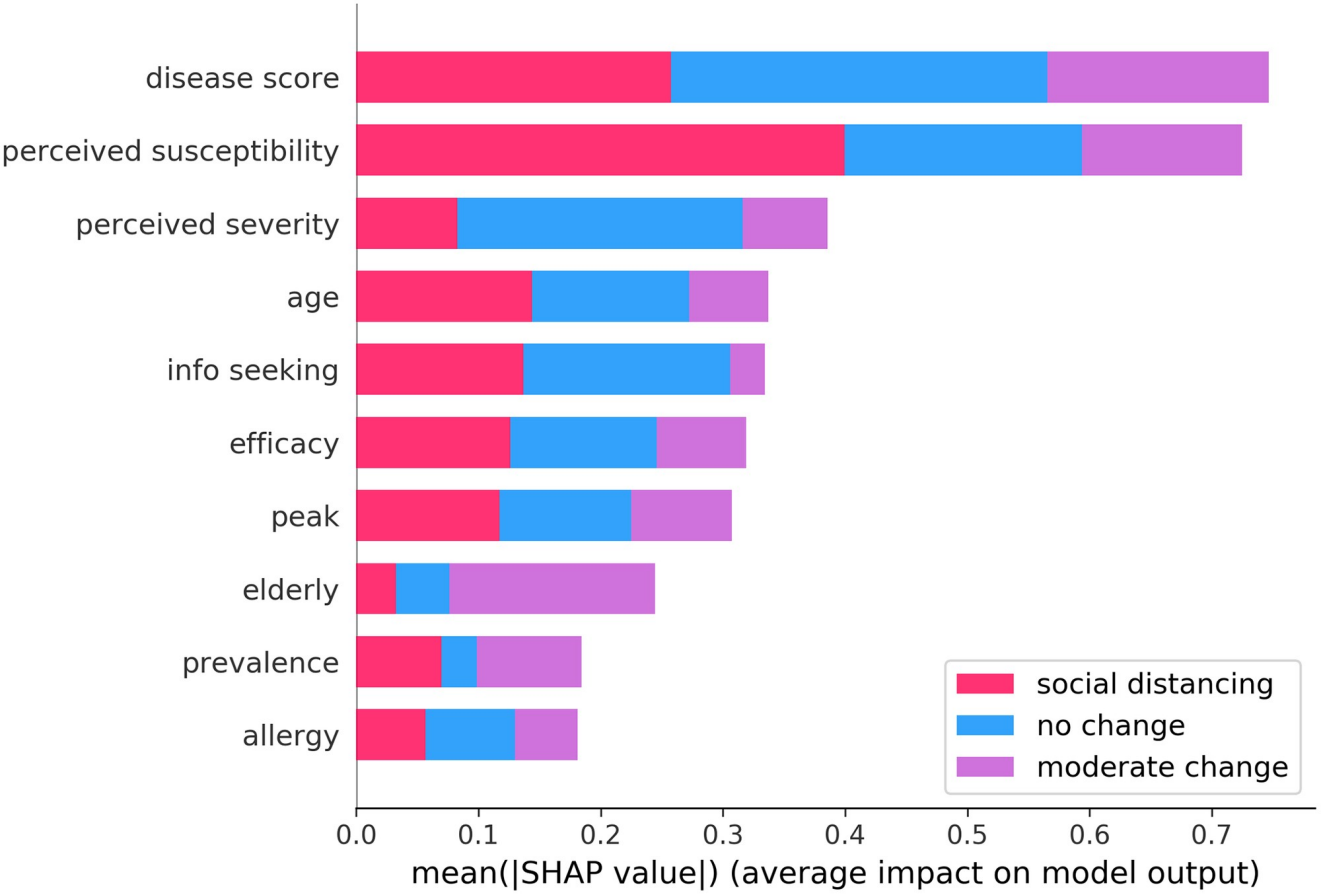
Summary plot for SHAP analysis. It shows the mean absolute SHAP value of ten most important features for the three classes.

In Fig 3 we have reported the mean absolute SHAP value of the ten most important features with respect to the three behavioral classes. This provides a general overview of the most influential features for the model and their impact on the classification of each behavioral class. Among the most determinant features we can recognize i) health-related factors (*disease score*, *allergy*) ii) personal beliefs (*perceived susceptibility*, *perceived severity*, *efficacy*) iii) socio-demographic indicators (*age*, *elderly*, *info seeking*), iv) information regarding the flu season (*peak*, *prevalence*). More in details, the top three features capture the severity and recency of past illnesses, the perceived susceptibility, and the perceived severity. Thus, having a history of illnesses induces users to be more careful and adapt their behaviors significantly. The last two features nicely match the HBM constructs associated to the drivers of behavioral changes. Notably, in seventh position we find the distance between the moment of response to the survey and the position of the peak. This suggests that the progression of the disease influences behaviors. Furthermore, seeking information about the flu has an important impact on the classification of class of no change and of social distancing. Thus, consulting news about the flu affects individuals’ decisions. Finally, it is interesting to notice how living with elderly people is significantly linked to moderate changes in behaviors. Users conscious of the risks of infection for elderly individuals might modify their behaviors as preventive measure. This highlights how behavioral changes are indeed a complex phenomenon possibly driven, at least in part, by altruistic concerns for others.

**Fig 4:**
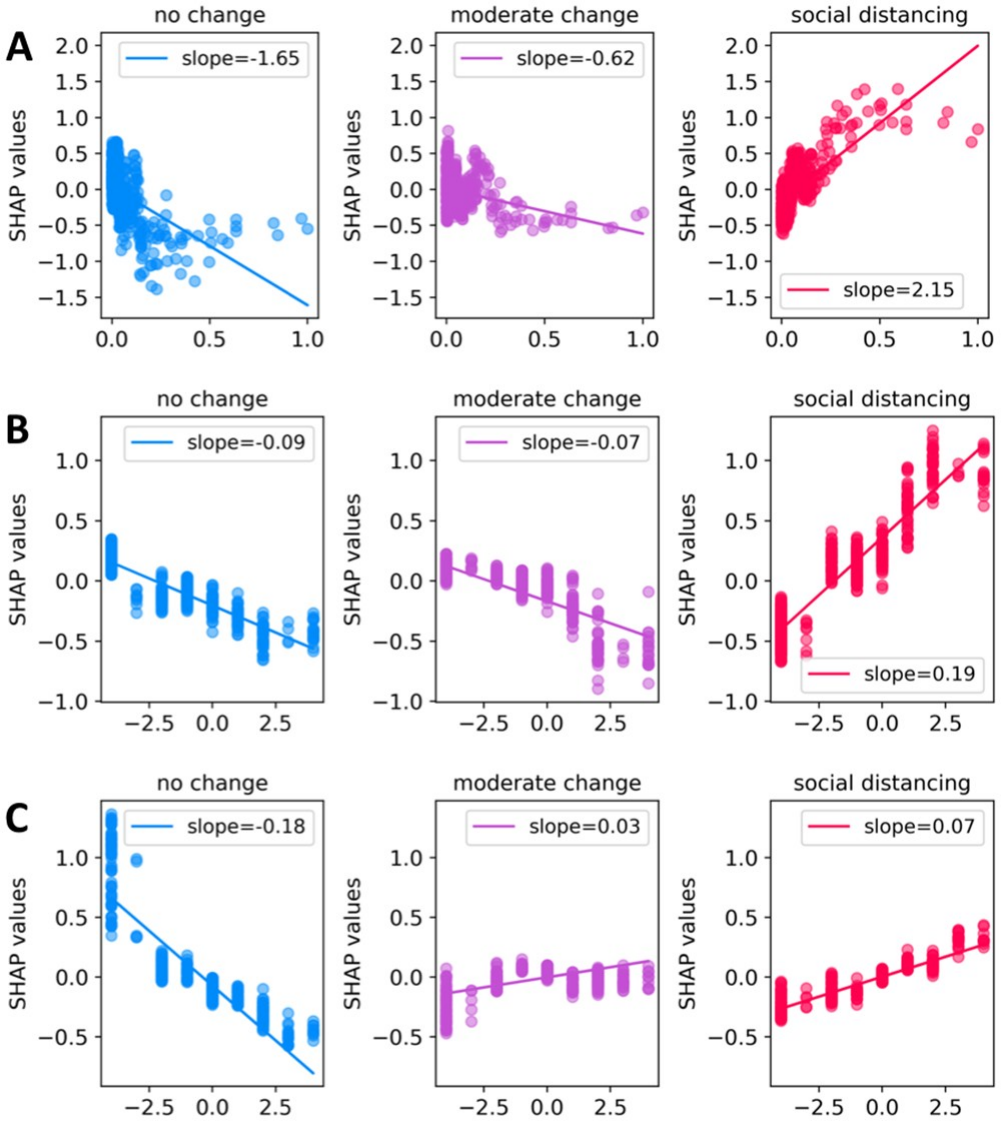
SHAP value plot for the three most important features: A) *disease score*, B) *perceived susceptibility*, C) *perceived severity*.

In order to deepen our understanding, in Fig 4 we study the relation between SHAP and features values for the three most important features: *disease score*, *perceived susceptibility*, and *perceived severity*. In particular, we plot the scatter plots in which the x-axes describe the feature and the y-axes the SHAP value for each of the 599 responses. In Fig 4A we inspect the effect of disease score on model’s decisions. We observe that having higher disease score has a huge positive effect on distancing measures. We observe that “low” and “medium” values of disease score have a positive non-negligible effect on the probability of adopting moderate behavioral measures. This observation is important to stress how a single variable might not be enough to capture the adoption of a particular behavioral category. In Fig 4B, we can observe that for the class of social distancing, SHAP values increase from negative to positive values as a function of *perceived susceptibility*. This suggests that those who perceive themselves as more susceptible to a possible contagion are more likely to adopt social distancing measures. Reasonably, for class of no change the trend of SHAP values is decreasing, meaning that those who do not feel susceptible will not probably change their behavior. The effects for the class of moderate change are similar to that of no change just discussed, even if we observe a weaker downward trend. In Fig 4C, we can observe that for the class of social distancing SHAP values increase from negative to positive values as a function of *perceived severity*. This prompts us to conclude that individuals who perceive the disease as more severe are also more likely to protect themselves through the adoption of social distancing measures. On the other hand, since for class of no change SHAP values show a decreasing trend, we can conclude also that those who perceive the disease as not particularly severe will not probably change any of their behaviors.

It is interesting to notice how these results are in line with empirical observations obtained, via telephone surveys, in China (Wuhan and Shanghai) during the COVID-19 pandemic [27]. In fact, moderate or severe anxiety has been found to be positively correlated with the perceived susceptibility and the perceived severity was the strongest predictor of behavioral changes [27]. Our findings are also in line with a preliminary account of surveys collected in Germany in early March 2020 [28]. In particular, the willingness to restrict people’s life was higher in case the underlaying reason was protecting vulnerable groups. Furthermore, our results provide insights for another study conducted in Europe during the early stages of the COVID-19 outbreak (i.e. before all top-down interventions) [26]. Respondents of online surveys, submitted via the InfluenzaNet network, have been found to be overly optimistic about the unfolding of the virus. The large majority of them estimated the risk of getting infected in the next two month to be lower or equal to one percent. Our findings suggest that these individuals were likely not to change in behaviors despite the news coming from China, evidence of local transmission in few municipalities in Italy and the recommendations of many health authorities. We can speculate that such low levels of perceived susceptibility and the resulting lack of behavioral changes at the early stages of the pandemic might have facilitated the spreading of the virus.

Overall, our observations are in very good accordance with what is assumed in the HBM regarding the existence of beliefs constructs—such as *perceived susceptibility* and *perceived severity*—that influence the probability of adopting safer behaviors. We can thus conclude that, not only the GBT model independently selects as fundamental drivers of behavioral change the belief constructs suggested in the HBM, but also that their effect is the same as theorized in the HBM. Furthermore, the result highlights the importance of personal past experiences with illnesses and altruism. From a public health perspective this suggests that, in order for communication campaigns to be more effective, they should leverage the individuals’ personal experience, stress how recommended behaviors might help achieve more positive personal health outcomes while protecting vulnerable populations.

### Conclusion

Our understanding of behavioral changes induced by infectious diseases is unfortunately extremely limited and anecdotal. The lack of data isolating and capturing this complex phenomenon is the key challenge. Here, we collected a unique dataset comprised of 599 responses to a questionnaire about behavioral changes submitted by *N* = 434 volunteers of the participatory Web platform Influweb during the 2017 − 18 and the 2018 − 19 flu seasons in Italy. For each response, we identified 23 features regarding socio-demographic information, personal history of illnesses, and sentiment about the flu epidemic, and one target class describing the type of self-initiated behavioral changes implemented by respondents. Then, we investigated the possibility of predicting these target classes from the features adopting a range of machine learning algorithms. Gradient Boosted Trees outperformed a random predictor, *SVM*, *Random Forest*, and *Logistic Regression*. While the average precision (across the three classes) of the best model is only 0.546, 66% of the samples belonging to the class of most drastic behavioral changes were correctly identified. It is important to notice how, to the best of our knowledge, there are not similar studies to compare and contrast our results. In fact, as mentioned above, the study of behavioral changes induced by disease has been mostly a theoretical endeavor.

Since we are interested in understanding the factors driving people to change behaviors, we investigated the patterns spotted by the GBT model. To this end, we exploited a recent tool in the field of explainable machine learning: SHAP. By using this approach we discovered that the intensity and the recency of past personal episodes of illnesses, perceived susceptibility and perceived severity of an infection are the most important features used for classification. These findings highlight the importance of negative past experiences and are in very good accordance with the expectations from the Health Belief Model. In fact, this theoretical framework predicates the existence of beliefs constructs (such as *perceived susceptibility* and *perceived severity*) as main drivers of behavioral change induced by epidemics. In the top ten of most important features we found also i) the timing of response in relation to the peak of the seasonal flu, ii) the extent to which participants sought information about the flu, iii) whether the participant was living with elderly people. These results suggest that indeed the progression of the season induces changes in behavior, that seeking information about the disease might affect individuals’ decisions and that individuals might change behaviors as a form of altruistic protection. In the SI, we verified the robustness and validity of our results by training the model on different subsets of our dataset and changing the definition of a key variable.

The presented study comes with limitations. First, while the socio-demographic indicators of participants are in line with the Italian population as a whole (see SI), the sample might be affected by self-selection biases. Indeed, we queried users willing to devote their time to the monitoring of the seasonal flu. Their sentiments, concerns and thus behaviors in response to the disease might deviate from those of the general population. Second, although, there are no other studies to compare the precision of our prediction task with, the absolute value of it is satisfactory, at best. The analysis of SHAP values and the connections of our findings with the well known theoretical constructs of the HBM are definitely reassuring. Nevertheless, future work is needed to collect larger samples and to quantify the general validity of these results. Third, we identified three classes of behavioral changes. Arguably, this classification could be refined to account for much more heterogeneity. To this end, the collection of larger samples is key. Finally, the study focuses only on one piece of the puzzle. Indeed, we did not investigate the effects of behavioral changes on the unfolding of the disease. Behaviors and diseases are linked by a feedback loop. Here, we simply focused on the first. Challenging future work is needed to connect these observations with the disease dynamics and back to the behaviors.

Overall, the research is a step towards the characterization of the factors driving behavioral changes during an outbreak. The study contributes to the small body of empirical literature on the subject and paves the way to future extensions and generalizations necessary to improve our understanding of human adaptive behaviors. From a public health stand point, a data-driven characterization of the key factors influencing changes in behaviors opens new perspectives to the possibility of predicting them and devising more effective communication campaigns aimed at mitigating transmission among individuals. In particular, our findings suggest that communication campaigns aimed at promoting safer/healthier behaviors should leverage personal past experiences and emphasize the altruistic component of such behaviors. As corollary, our results indicate that public health officials might face harder challenges to promote changes in behaviors in case of novel diseases, especially during the earliest phases of their spread. In fact, the lack of personal experiences might exacerbate the optimistic bias and result in an underestimation of perceived susceptibility and severity [75, 76]. This observation is in line with empirical observations from China and Europe during the COVID-19 pandemic [26–28]. Indeed, while in the epicentre of the outbreak changes in behaviors were strongly linked to perceived severity, in Europe, during the earliest phases of the pandemic, the large majority of individuals considered themselves at very low risk despite the news from China and the indications from public health officials. Arguably, these individuals were likely not to implement self-initiated changes in behaviors as way to reduce transmission. The research presented here suggest that in these cases communication strategies could leverage the altruistic benefits of adaptive behaviors.

Finally, as we write the COVID-19 pandemic is sweeping across the globe with unprecedented socio-economical costs. As result, despite initial phases of reluctancy, governments have taken strict top-down measures aimed at hampering the spreading. While the focus on this paper is on milder, yet significant, diseases that do not induce such strong reactions, our results are in line and provide insights to interpret the preliminary work prompted by the current health emergency [26–28]. More in general, this pandemic has dramatically exposed our unpreparedness to deal and cope with new emerging diseases. In the specific context of the research presented here, COVID-19 has highlighted the importance of understanding and modeling behavioral changes to improve the predictive power of epidemic models, design more efficient communication strategies aimed at nudging populations towards safer behaviors, and characterize the adaptive nature of human behavior. It is early to speculate about the long lasting impact of COVID-19 in our society, but arguably these unprecedented events will leave a deep scar that might affect the perceived susceptibility and severity, hence our behaviors, of future outbreaks. The results presented in this paper, obtained in a pre COVID-19 world, offer the possibility to quantify the impact of the current pandemic after its course. Moreover, this work was conceived in a very different historical moment with a focus on seasonal flu but the relevance of its approach and the importance of surveying the general population to collect disease and behavioral information that would be otherwise not accessible to health authorities have become even more evident during the present unprecedented conditions. Supporting and strengthening already established participatory surveillance platforms could help boosting our preparedness to future outbreaks.

## Supporting information

S1 Appendix In this appendix we report some qualitative observations on the data and we compare our sample to the Italian population as a whole in terms of age, gender and geographic distribution. We provide a brief description of the classification algorithms that we used, the specs, and the codes. We present an example to show how SHAP helps us in understanding model’s decisions. We assess the stability of GBT classification results training the model on various subsets of the dataset. In particular, we consider singularly the two flu seasons, we consider a single survey for each user, we disregard behavioral features, and finally we consider a simplified definition of the disease score.
(PDF)
