# Supplementary material for: Towards a data-driven characterization of behavioral changes induced by the seasonal flu
Source: PLoS Comput Biol. 2020 May 13;16(5):e1007879. doi: 10.1371/journal.pcbi.1007879 (PMC7250468; doi:10.1371/journal.pcbi.1007879)
Supplement: S1 Appendix — We provide a brief description of the classification algorithms that we used, the specs, and the codes. We present an example to show how SHAP helps us in understanding model’s decisions. We assess the stability of GBT classification results training the model on various subsets of the dataset. In particular, we consider singularly the two flu seasons, we consider a single survey for each user, we disregard behavioral features, and finally we consider a simplified definition of the disease score. (PDF) [file pcbi.1007879.s001.pdf]

Towards a data-driven characterization of behavioral changes induced by the seasonal flu

Nicolò Gozzi[1, *], Daniela Perrotta [2], Daniela Paolotti[3], Nicola Perra[1, 3]

1 Networks and Urban Systems Centre, University of Greenwich, London, UK
2 Max Planck Institute for Demographic Research, Rostock, Germany
3 ISI Foundation, Turin, Italy

* N.Gozzi@gre.ac.uk

S1 Appendix

Descriptive analysis of data

We report here a few qualitative observations on the data.

First, responses are divided in the three behavioral categories as follows: 26% report no changes, ii) 36% only moderate changes, iii) 38% significant variations. Thus the majority of them describe either moderate or severe changes in behaviors.

Second, only 20% of the participants that reported variations in their behaviors in a particular season, experienced the flu in that season before or while compiling the survey. We identify the cases of flu using the definition of the European Centre for Disease Prevention and Control (ECDC) [1]. According to this definition, seasonal flu is identified by the following criteria: sudden onset of symptoms, at least one systemic symptom (fever or feverishness, malaise, headache, myalgia), and at least one of respiratory symptom (cough, sore throat, shortness of breath).

Third, during $2017-18$ season, 27% of the answers provided before the peak are related to the category of no change, 46% to moderate change and 27% to social distancing. After the peak, instead, the number of surveys related to no change is 23%, to moderate change is 34%, while the percentage of surveys related to social distancing significantly grows up to 43%. Very similarly, during $2018-19$ season before the peak we have 29% of surveys that report any change in behavior, 39% that report only moderate change to behavior, and 32% that report the adoption of social distancing measures. The division after the peak, instead, is the following: 26% of surveys related to no change, 29% to moderate change and 45% to social distancing (Fig A). Interestingly, in both seasons we note a significant increase in the number of surveys reporting social distancing measures after the peak. This observation highlights how risk perception is linked to the progression of the disease and not constant throughout. Indeed, proactive behavioral measures adopted by people seem to strengthen when external conditions get worse.

Fourth, the comparison of our sample with the statistics retrieved from the online archive of the Italian National Institute of Statistics (ISTAT) [2] in Fig B shows that the geographic, gender and age distributions of participants are almost in line with the Italian population as a whole. In particular, we note a slight over representation of i) Northern Italy (+13% north west, +7% north east), ii) of male (+11%), and iii) of age group 15-64 (+12%). In Fig C we compare also our sample to the general population in terms of education level. In this case we observe a significant over-representation of individuals with a higher level of education (+33%). We know from previous research

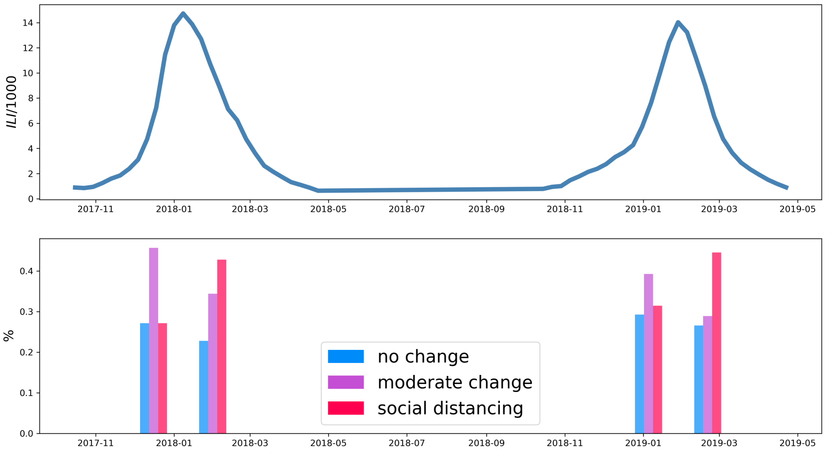

Fig A. Flu incidence in the two seasons (above) and behavioral class distribution before/after the peak (below). The two seasons had a similar impact in terms of epidemic incidence, while the peaks are slightly shifted (2nd week of 2018 for the 2017/18 flu season, and 5th week of 2019 for the 2018/19).

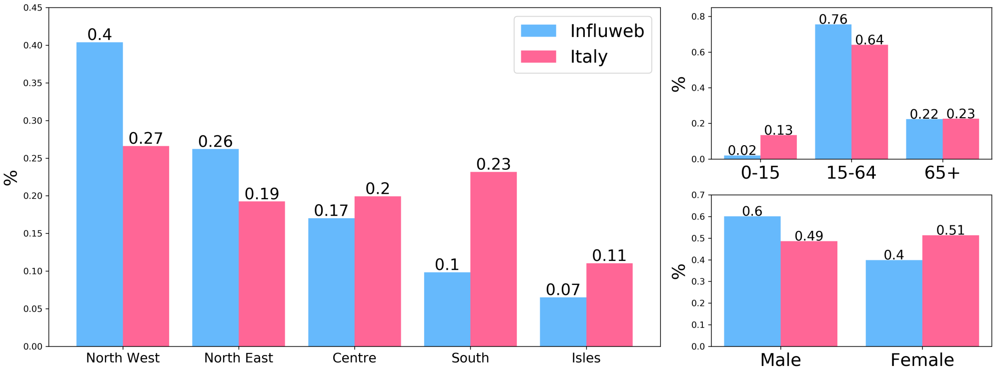

Fig B. Comparison of our sample with the general Italian population in terms of age, gender and home area.

that this can be an issue associated with internet-based studies [3–5]. The representativeness of Influweb - the platform we used to collect data - has been addressed in more detail in [6]. Unfortunately, we do not have information regarding the level income of participants. However, previous research on participants of Flu Near You and of other Influenzanet campaigns showed that their socioeconomic status tends to be higher than he general populations in their respective countries [7–9].

Finally, in table A we list the behaviors changed by individuals in the class of *social distancing* and in the class of *moderate change*. As an example, we classify in the class

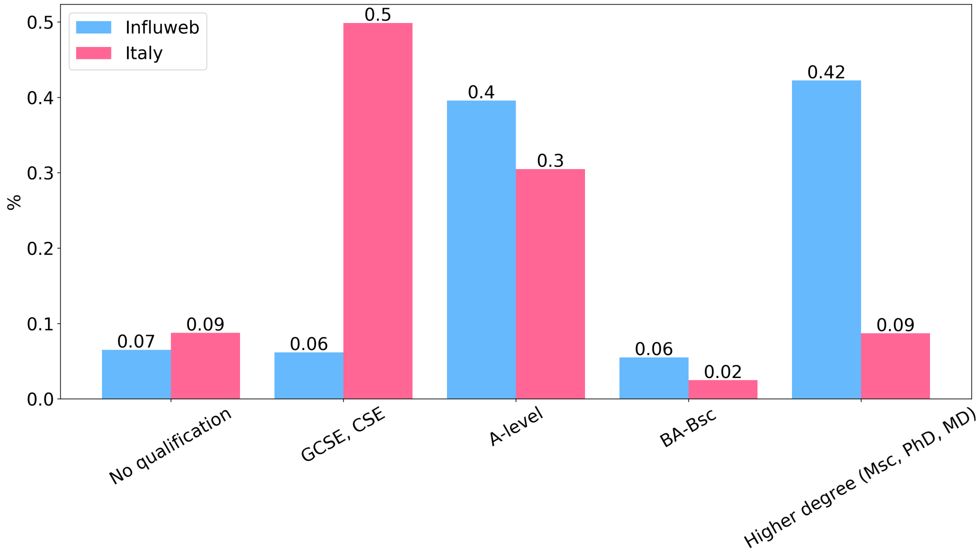

Fig C. Comparison of our sample with the general Italian population in terms of education level.

of *social distancing* an individual who responded both "Reduced the amount you go to school/work" and "Reduced the amount you touch your eyes, nose, or mouth", while we classify in the class of *moderate change* an individual who responded both "Cleaned or disinfected things you might touch more often" and "Usually carried tissues with you when out more often".

Table A. Behaviors changed in *social distancing* and *moderate change* classes.

**social distancing**	**moderate change**
Reduced the amount you go to school/work	Cleaned or disinfected things you might touch more often
Cancelled or postponed social events such as meeting friends	Carried sanitising hand gel when going out more often
Reduced or changed the way you use public transportation	Used sanitising hand gel to clean your hands more often
Reduced the amount you go into shops	Reduced the amount you touch your eyes, nose, or mouth
Kept one or more of your children out of school or nursery	Followed a healthy diet or took vitamin supplements more often
Kept away from crowded places	Usually carried tissues with you when out more often
Tried to avoid people who have flu	Usually used tissues when sneezing or coughing more often

Classification algorithms

Decision tree ensembles are a powerful tool for classification tasks [10]. They consist in a set of classification trees. The underlying idea is that summing together the predictions of multiple "weak" learners, one can achieve more robust predictions than with a single "strong" learner. Mathematically, the prediction $\hat{y}_i$ of this model for the *i-th* point of the dataset can be written as:

$$\hat{y}_i = \sum_{k=1}^{K} f_k(x_i) \tag{1}$$

Where K is the number of trees (i.e. the size of the ensembles) and $f_k(x_i)$ is the prediction of the *k-th* tree for the *i-th* point. This general model is implemented by a

great variety of algorithms, such as Gradient Boosted Trees (GBT) [11] and Random Forest (RF) [12]. The differences arise from how trees are built and added. GBT exploits a specific training strategy called *additive training*, in which at each training step is added to the ensemble the tree that optimizes the objective function. Recently, GBT has gained a great popularity for its speed and performance, and has become the algorithm of choice for many machine learning applications.

Support Vector Machines (SVM) [13] extends the concept of Linear Classification by trying to find a hyperplane in an N-dimensional space (where N is the number of features) that distinctly classifies the data points and that has the maximum margin. This approach is more stable to noise and to the classification of future points. However, one of the main advantages of SVM is the so-called "kernel trick". In fact, using this method SVM can easily handle also seriously non-separable data. Through the kernel trick data can be easily projected in a vector space where they are - eventually - linearly separable. The only request for this new space is that the dot product (called *kernel*) must be defined. Commonly used kernels are the polynomial kernel or the radial basis function kernel (RBF). This approach makes SVM a very powerful tool that can be exploited to classify a broad range of datasets.

Logistic Regression (LG) [14] is a very common tool used to predict probabilities. Given a sample input vector $x \in \mathbb{R}^n$ and the vector of model's weights $\theta \in \mathbb{R}^n$, LG smoothly projects the signal $s = \theta^T x$ in the probability range $[0, 1]$ using - for example - a logistic function defined as:

$$\gamma(s) = \frac{e^s}{1 + e^s} \tag{2}$$

The output can then be interpreted as a probability for a binary event. This framework can be easily extended to the cases with more than two possible outcome.

Dummy Predictors (RND) are generally used as null benchmarks to assess whether the models learnt something from data. There are many trivial prediction strategies, for example constant or equally probable predictions ("coin toss"). In this work we consider a random predictor that generates random predictions by respecting the training set's class distribution.

For all the models we use open-source implementation. In particular, we use *XGBoost*, an open-source software library which provides a "scalable, portable and distributed implementation" of GBT [15, 16]. For other models we use the implementation provided by the open-source library *scikit-learn* [17] and we train them fine-tuning standard parameters. The source code can be find at [18].

Sensitivity analysis

We test here the stability of classification results of GBT. To this end, we train the model on various subsets of the whole dataset. First, we classify separately the surveys submitted during the $2017-18$ ($N = 331$ surveys) and the $2018-19$ ($N = 268$ surveys) flu season. We obtain a *precision* of 0.572 for the first and of 0.474 for the second season. These results are still significantly higher than the random prediction and are comparable to those obtained in the general case with the two seasons aggregated ($precision = 0.546$). Second, we repeat the classification excluding from the set of features those extracted from behavioral surveys. The goal of this analysis is to explore the possibility of inferring attitudes towards behavioral change on a much bigger scale. In fact, behavioral questionnaires represent an additional burden for the participants and mostly important Influweb is one of 9 platforms in Europe. As reported in table B, disregarding features deriving from behavioral surveys leads to a *precision* of 0.435. Despite an expected decrease in model's performance, results are still better than the random guess. These observations are encouraging and shed light on the possibility of

extending of our approach. However, larger samples as well as data from other countries are needed to reinforce and scale our findings. Third, we consider a much simpler definition of the most important feature used in classification: *disease score*. In particular we consider the average number of symptoms reported by different individuals in symptoms questionnaires as a measure of severity of past illnesses. In other words, we disregard the exponential temporal weights of the original definition. Looking at results in table B, we note that the adoption of this simpler definition of *disease score* does not significantly affect results, even if the classification performance across the four metrics is slightly lower. Fourth, we repeat the analysis considering only one survey for each user (we consider the most recent ones). In table B we can see that the classification performance is still higher than the random benchmark. Finally, we consider 2 behavioral classes instead of 3. The problem then become a binary classification between the class of *no change* and that of *any change*. We report a *precision* of 0.743 with respect to 0.618 of the random prediction. Notably, the five most important features in the *unique* and *2 classes* cases are still those emerged in the complete 3-class problem (*perceived susceptibility*, *disease score*, *info seeking*, *perceived severity* and *age*).

Table B. GBT sensitivity analysis.

	**precision**	**bal. accuracy**	**recall**	**f1 score**
RND	0.343	0.335	0.334	0.335
2017/18	0.572	0.556	0.550	0.553
2018/19	0.474	0.455	0.444	0.441
no behavioral survey	0.435	0.432	0.439	0.435
simplified disease score	0.530	0.533	0.528	0.524
unique	0.478	0.461	0.473	0.472
2 classes	0.743(0.618)	0.646(0.504)	0.761(0.503)	0.748(0.532)

SHAP, an example

In SHAP, the classic definition of the Shapley value is adapted to the specific problem of assigning to each feature its importance in the classification task. The result is called SHAP value and, for feature i, is defined as:

$$\phi_i = \sum_{S \subseteq N \setminus \{i\}} \underbrace{\frac{|S|!(M-|S|-1)!}{M!}}_{(1)} \underbrace{[p(S \cup \{i\}) - p(S)]}_{(2)} \tag{3}$$

Where, S are all the possible subsets of features without i; $|S|$ is the number of features in S; M is the total number of features; $p(S \cup \{i\})$ is the outcome of prediction considering both i and features in S, and $p(S)$ is the outcome considering only features in S. To better understand the meaning of ϕ_i, we divide its expression in blocks. In expression 3, part (1) is an interaction term that accounts for all possible sequences in which features are added: in fact, the order in which features are taken into account is meaningful to how their importance is assigned. Given this, we move to part (2). Intuitively, if feature i has a negligible impact on model's decisions, then considering it or not should not affect the prediction. Looking at 3 this is equivalent to saying:

$$p(S \cup \{i\}) \simeq p(S) \rightarrow \phi_i \simeq 0 \tag{4}$$

On the other hand, if feature i is decisive for the classification we expect $p(S \cup \{i\}) \neq p(S)$ and $\phi_i \neq 0$. As a guiding criterion, if feature i is *important* for the

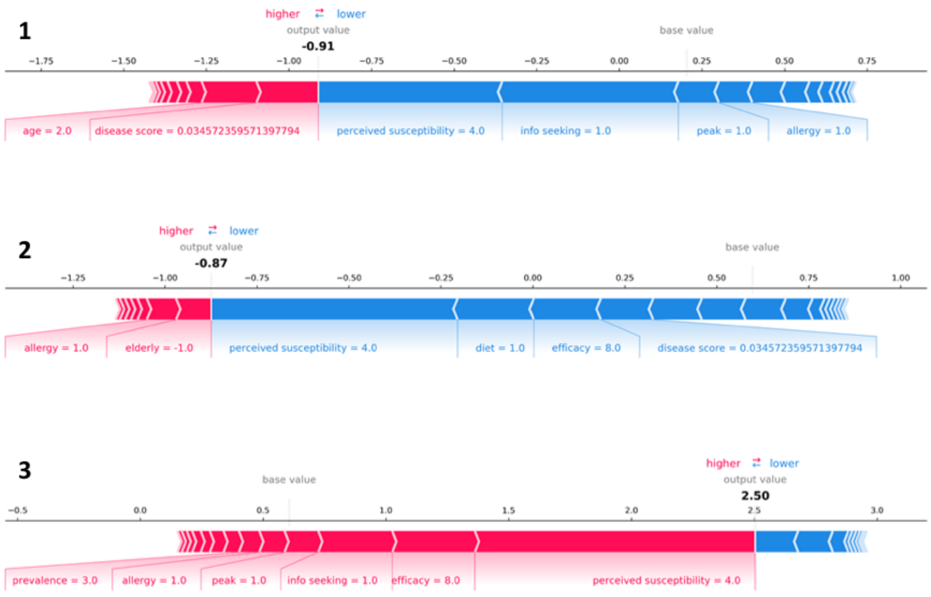

Fig D. SHAP example: 1) is related to no change, 2) to moderate change, and 3) to social distancing class.

classification of an individual then its SHAP value will be significantly different from zero. More in detail, if it is negative, the feature under consideration lowers the likelihood of belonging to that particular class, if it is positive it raises it.

In this appendix we present also an example on how SHAP works and how it helps in understanding model's decisions. In Fig D we try to explain the class prediction for a specific individual made by GBT. The chart is composed of three figures and each one refers to a specific class: D1 is related to the no change class, D2 to moderate change, and D3 to social distancing. The individual under examination belongs to the class of social distancing and is classified correctly. This can be figured out examining the output of the model for each class: -0.91 for no change class, -0.87 for moderate change, and 2.50 for social distancing. Intuitively, these numbers indicate that the most unlikely class for this individual is no change. A little bit more likely is moderate change, but the class of social distancing is far more probable. However, SHAP provides us much more information. In fact, we can evaluate the role played by each feature in the classification: the blue ones in figure have a negative effect on the likelihood of belonging to that particular class, while the red ones have a positive effect. Furthermore, the length of the arrow (red or blue) related to each feature represents its SHAP value. Then, for example, we can conclude that for this individual, *info seeking* and *perceived susceptibility* both have an important negative impact on the likelihood of being in the no change class. Conversely, they have a great positive impact on the likelihood of being in the correct class, social distancing. Finally, we can give an interpretation of the prediction: since the individual feels vulnerable to the disease (*perceived susceptibility*$= 4$) and searches regularly for information regarding the flu (*seek*$= 1$), then she is with great confidence a member of the class of social distancing.

References

1. EU case definitions - Influenza;. https://www.ecdc.europa.eu/en/surveillance-and-disease-data/eu-case-definitions.

2. Istat Online Archive;. https://www.istat.it/it/archivio/demografia.

3. COUPER MP. Review: Web Surveys: A Review of Issues and Approaches*. Public Opinion Quarterly. 2000;64(4):464–494. doi:10.1086/318641.

4. Hartz S, Quan T, Ibiebele A, Fisher S, Olfson E, Salyer P, et al. The significant impact of education, poverty, and race on Internet-based research participant engagement. Genetics in medicine : official journal of the American College of Medical Genetics. 2016;19. doi:10.1038/gim.2016.91.

5. Gosling SD, Vazire S, Srivastava S, John OP. Should we trust web-based studies? A comparative analysis of six preconceptions about internet questionnaires. The American psychologist. 2004;59 2:93–104.

6. Cantarelli P, Debin M, Turbelin C, Poletto C, Blanchon T, Falchi A, et al. The representativeness of a European multi-center network for influenza-like-illness participatory surveillance. BMC public health. 2014;14:984. doi:10.1186/1471-2458-14-984.

7. Smolinski M, Crawley A, Olsen J, Jayaraman T, Libel M. Participatory Disease Surveillance: Engaging Communities Directly in Reporting, Monitoring, and Responding to Health Threats. JMIR Public Health and Surveillance. 2017;3:e62. doi:10.2196/publichealth.7540.

8. Bajardi P, Vespignani A, Funk S, Eames K, Edmunds W, Turbelin C, et al. Determinants of Follow-Up Participation in the Internet-Based European Influenza Surveillance Platform Influenzanet. Journal of medical Internet research. 2014;16:e78. doi:10.2196/jmir.3010.

9. Baltrusaitis K, Santillana M, Crawley A, Chunara R, Smolinski M, Brownstein J. Determinants of Participants' Follow-Up and Characterization of Representativeness in Flu Near You, A Participatory Disease Surveillance System. JMIR Public Health and Surveillance. 2017;3:e18. doi:10.2196/publichealth.7304.

10. Gashler M, Giraud-Carrier C, Martinez T. Decision Tree Ensemble: Small Heterogeneous Is Better Than Large Homogeneous. In: 2008 Seventh International Conference on Machine Learning and Applications; 2008. p. 900–905.

11. Friedman JH. Greedy Function Approximation: A Gradient Boosting Machine. The Annals of Statistics. 2001;29(5):1189–1232.

12. Breiman L. Random Forests. Mach Learn. 2001;45(1):5–32. doi:10.1023/A:1010933404324.

13. Suykens JAK, Vandewalle J. Least Squares Support Vector Machine Classifiers. Neural Process Lett. 1999;9(3):293–300. doi:10.1023/A:1018628609742.

14. Yarnold P, Grimm L. Reading and Understanding Multivariate Statistics; 1995.

15. Chen T, Guestrin C. XGBoost: A Scalable Tree Boosting System. CoRR. 2016;abs/1603.02754.

16. XGBoost repository;. https://github.com/tqchen/xgboost.

17. Pedregosa F, Varoquaux G, Gramfort A, Michel V, Thirion B, Grisel O, et al. Scikit-learn: Machine Learning in Python. Journal of Machine Learning Research. 2011;12:2825–2830.

18. Source code;. https://github.com/ngozzi/behavioralchange.
